# Evaluating large language models in biomedical data science challenges through a classroom experiment

**DOI:** 10.1073/pnas.2521062122

**Published:** 2025-12-11

**Authors:** Huifang Ma, BIOSTAT 824 Student Consortium, Zhicheng Ji

**Affiliations:** ^a^Department of Biostatistics and Bioinformatics, Duke University School of Medicine, Durham, NC 27705

**Keywords:** large language model, data science, machine learning

## Abstract

Large language models (LLMs) have shown remarkable capabilities in algorithm design, but their effectiveness in solving data science challenges in real-world settings remains poorly understood. We conducted a classroom experiment in which graduate students used LLMs to solve biomedical data science challenges on Kaggle, focusing on tabular data prediction. While their submissions did not top the leaderboards, their prediction scores were often close to those of leading human participants. LLMs frequently recommended gradient boosting methods, which were associated with better performance. Among prompting strategies, self-refinement, where the LLM improves its own initial solution, was the most effective, a result validated using additional LLMs. While LLMs are capable of handling more complex data science tasks beyond tabular data prediction, their performance is substantially worse. These findings demonstrate that LLMs have the potential to design competitive machine learning solutions, even when used by nonexperts.

## Significance

Large language models (LLMs) are increasingly used in science and engineering, yet their real-world effectiveness in data analysis remains unclear. In this study, graduate students used LLMs to tackle biomedical data challenges on Kaggle, a popular data science platform. Despite limited programming input, the LLM-guided solutions performed surprisingly well, often nearing top-performing human entries. The most effective strategy was self-refinement, where the LLM iteratively improves its own code. This work shows that LLMs can help nonexperts generate competitive machine learning solutions, highlighting their potential to broaden access to data science and accelerate research across disciplines.

Large language models (LLMs) are advanced deep learning architectures trained on vast corpora of textual data to understand and generate human-like language. These models, such as GPT (1), BERT (2), and their successors, leverage transformer-based architectures to capture complex linguistic patterns, semantic relationships, and contextual dependencies across long sequences of text. LLMs have demonstrated remarkable performance across a wide range of natural language processing tasks. Their generalization ability, combined with scalability through transfer learning and prompt engineering, has positioned LLMs as foundational tools in modern artificial intelligence research and applications.

LLMs have emerged as powerful tools for code generation, transforming natural language instructions into syntactically valid and semantically meaningful code across a variety of programming languages. Early advances were driven by models such as OpenAI’s Codex (3), which demonstrated the ability to solve around 28.8% of Python problems in the HumanEval benchmark when prompted with a single solution, and up to 70% with multiple samples, outperforming GPT-3 by a significant margin. Building on this foundation, AlphaCode by DeepMind achieved human-level performance in programming contests, placing within the top 54.3% on Codeforces and demonstrating the potential of LLMs to solve complex, competitive tasks (4). Meta AI’s InCoder (5) and CodeGen (6) further advanced LLM capabilities by enabling code infilling and supporting multilingual programming, thereby improving usability for real-world development. SantaCoder (7) introduced efficient, open-access training for multilingual code generation. StarCoder (8), trained on over 1 trillion tokens of code, showed strong performance across multiple benchmarks while remaining accessible for academic use. Code Llama, Meta’s most recent release, improved on previous models in both zero-shot and few-shot settings (9), while WizardCoder (10) applied instruction tuning to further enhance accuracy. In addition, LLMs like PanGu-Coder2 (11) and DeepSeek-Coder (12) have demonstrated that instruction tuning, multiround prompting, and domain-specific adaptation can yield substantial improvements, with some open-source models approaching GPT-4-level performance in controlled benchmarks. Collectively, these findings highlight not only the rapid evolution of LLMs for code generation but also their increasing accessibility, efficiency, and utility in real-world programming tasks. More recent evaluations confirm that state-of-the-art models can match or surpass most human programmers. For example, GPT-4 was found to outperform approximately 85% of participants on LeetCode and GeeksforGeeks coding challenges when optimally prompted (13).

Beyond generating code for algorithmic tasks, LLMs have also been applied to data science challenges that require constructing end-to-end analytical pipelines using real-world data (14). Several benchmark studies systematically evaluate LLM performance and demonstrate competence across core data science tasks (15–17). Building on these capabilities, LLM-powered agents are emerging as full-lifecycle systems that plan analyses, generate and execute code, inspect results, and iteratively refine outputs through self-reflection and tool use (18, 19). Noteworthy examples include Data Interpreter, which employs graph-based planning (20), DS Agent, which applies case-based reasoning (21), and DatawiseAgent, which integrates tightly with notebooks (22), while production-ready agents such as Data Copilot (23) and AgentAda (24) adapt to varying skill levels and operational needs. Complementary frameworks like AutoGen (25) and DSPy (26) support this ecosystem by compiling prompt-driven workflows into reproducible, optimizable pipelines.

A limitation of existing research concerns the nature of the evaluators themselves. Most benchmarking studies are conducted by researchers who are highly familiar with LLMs, many of whom are experts in prompt engineering or directly involved in LLM development. These individuals tend to craft carefully optimized prompts that leverage deep knowledge of model behavior, often resulting in inflated performance metrics that do not generalize to typical users. In contrast, the broader population of users, such as data scientists, students, or professionals in applied domains, often lack specialized training in how to interact effectively with LLMs. As a result, the actual performance of LLMs when used by nonexperts may differ substantially from what published benchmarks suggest. Another limitation is that existing studies only compare performance across various LLMs or AI agents, but lack a clear understanding of how LLMs perform relative to human data scientists. Such comparisons are essential for evaluating the actual progress and limitations of contemporary LLMs in real-world, end-to-end analytical tasks. They help identify which areas of data science LLMs can effectively automate and deliver results comparable to those produced by human experts, and which areas still require human judgment, domain knowledge, or creative reasoning. These insights not only guide the development of human–AI collaboration strategies in practical environments but also inform how we educate the data science workforce, highlighting opportunities to leverage LLMs as learning tools that can enhance, rather than replace, human skill development. In summary, while existing research emphasizes method development and benchmark optimization, it fails to address the critical challenge of evaluating and integrating LLMs into real-world data science workflows.

In this study, we present the results of a classroom experiment in which 33 graduate students enrolled in a data science course were tasked with solving biomedical data science challenges published on Kaggle using OpenAI’s LLMs. We compared the performance of LLM-assisted students with that of human data scientists who participated in the same Kaggle competitions. Additionally, we investigated factors that contribute to the improved performance of LLMs in solving data science problems. Our findings show that the performance of LLMs, when prompted by graduate students, is comparable to that of the majority of human participants. Notably, we found that the use of self-refinement prompting strategies was effective in enhancing model outputs. When faced with more complex data science tasks, LLMs tend to perform substantially worse, likely due to their limited reasoning capabilities and lack of domain-specific knowledge. This study fills a critical gap by systematically evaluating how nonexpert users leverage large language models to tackle real-world data science challenges, providing insights into model usability, practical performance, and the evolving role of LLMs in applied data science workflows.

## Results

### Experiment Design.

The classroom experiment was conducted with 33 graduate students enrolled in the course BIOSTAT 824: Case Studies in Biomedical Data Science, offered by the Department of Biostatistics and Bioinformatics at the Duke University School of Medicine during the Spring 2025 semester. As the final project, students were tasked with using OpenAI’s o1 or o3 language model to solve six biomedical data science challenges hosted on Kaggle, a widely used platform for data science competitions. To accommodate the limited computational resources accessible to the students, all six challenges were selected from Kaggle’s “Tabular Playground Series,” where the training datasets are provided in a lightweight, tabular format. All challenges were published online on or after October 2023, which is the knowledge cutoff of the o1 and o3 models, and are therefore unlikely to have been included in their training data. To simulate a scenario in which users lack programming expertise, students were restricted to using only natural language prompts when interacting with the LLM. To prevent LLMs from retrieving answers online, students were required to disable the web search function of the OpenAI models. Each student was required to submit the complete chat history and the final code generated for each challenge as part of their project deliverables. The detailed instructions for the final project and the six Kaggle data science challenges are provided in *Methods*.

We then ran the final code submitted by the students to generate the prediction files, submitted these files to the Kaggle website, and recorded the corresponding prediction performance. This performance was compared to that of all human data scientists listed on the leaderboard, and a percentile rank was calculated. Students were graded based on their aggregated performance across all six tasks (*Methods*). Details of the students’ performance and the analysis of prompt messages are provided in *SI Appendix*, Table S1.

### Performance Overview.

**Fig. 1.**
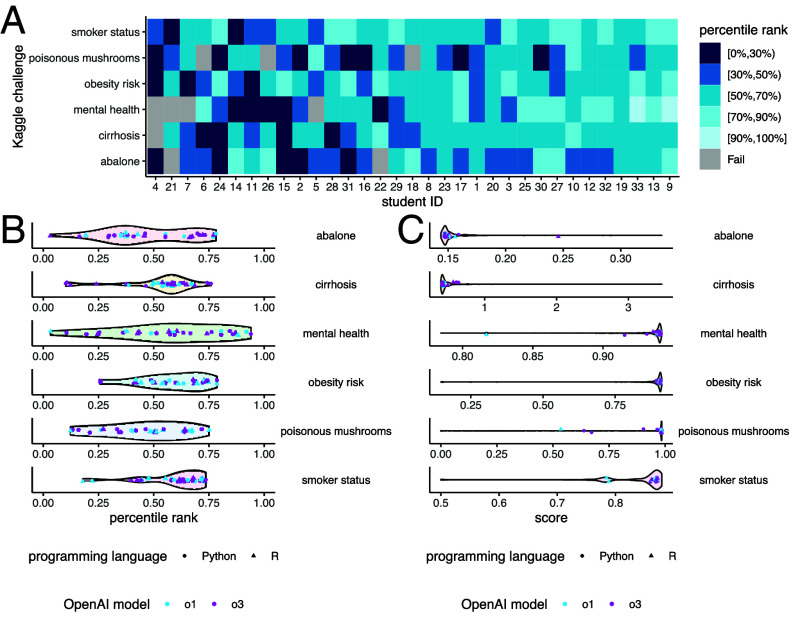
Performance overview. (*A*) Kaggle leaderboard percentile rank for each student and each Kaggle challenge. Gray indicates solutions that failed to pass Kaggle’s automatic scoring system. (*B*) Violin plots showing the percentile ranks of all student solutions for each Kaggle challenge. Each colored data point represents a single student’s solution. (*C*) Violin plots showing the scores of all human participant solutions recorded on the Kaggle leaderboard for each challenge. Each colored data point represents a student’s solution. Note that the violin plots in (*B* and *C*) represent different populations.

Fig. 1*A* shows the evaluation results of all LLM-generated solutions across six Kaggle challenges. In 95% of cases, we successfully generated valid prediction files that passed the Kaggle evaluation system based on the code submitted by the students. In the remaining 5% of cases, the code either contained programming errors or produced invalid prediction files that failed to pass the evaluation. These results suggest that LLMs are highly reliable for real-world applications, as they can autonomously generate functional code that meets external evaluation standards in the vast majority of cases. LLM performance across the six challenges demonstrated a general trend of consistency, with students who performed well on one challenge also tending to perform well on others.

Fig. 1*B* shows the distribution of percentile ranks for the LLM-generated solutions. The majority of solutions achieved a percentile rank between 25% and 75% across all six challenges. There were no substantial differences in performance between solutions generated using different OpenAI models (o1 or o3) or programming languages (R or Python). Fig. 1*C* compares the distribution of performance scores for all human data scientists listed on the Kaggle leaderboard with those of the LLMs. Notably, the absolute performance of the top-performing human data scientists is tightly clustered, meaning that even minor differences in prediction scores can lead to large shifts in percentile rank. As a result, while LLMs’ scores were often very close to those of the best-performing models, their percentile rankings could vary significantly. Nonetheless, most LLM solutions were comparable in accuracy to those of a high-performing subgroup of human data scientists. These results demonstrate that LLMs can generate solutions that are practically effective for a variety of biomedical data science tasks, even if they do not top the leaderboard.

### Machine Learning Strategies.

**Fig. 2.**
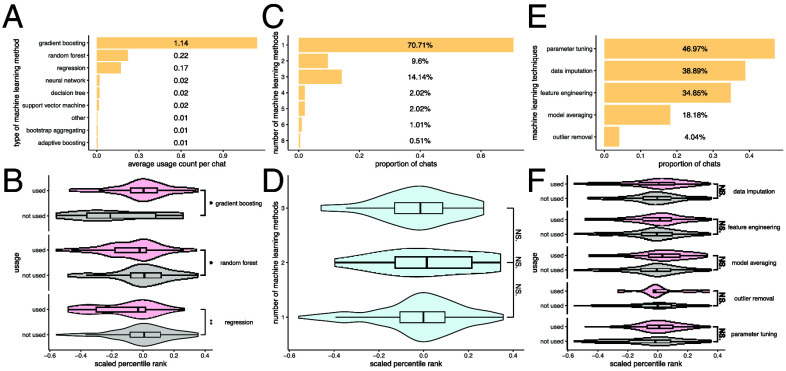
Machine learning strategies. (*A*) Average number of uses per chat for different types of machine learning methods. (*B*) Distribution of scaled percentile rank comparing solutions that did or did not use a given type of machine learning method. Wilcoxon tests were performed to compare the two distributions. “**” indicates a *P*-value between 0.001 and 0.01, and “*” indicates a *P*-value between 0.01 and 0.05. (*C*) Proportion of chats that used different numbers of machine learning methods. (*D*) Distribution of scaled percentile rank comparing solutions that used different numbers of machine learning methods. Wilcoxon tests were performed to compare the distributions. “NS.” indicates the *P*-value is not statistically significant. (*E*) Proportion of chats that used different machine learning techniques. (*F*) Distribution of scaled percentile rank comparing solutions that did or did not use a given machine learning technique. Wilcoxon tests were performed to compare the distributions. “NS.” indicates the *P*-value is not statistically significant.

We manually analyzed the final submission code generated by LLMs and curated the machine learning methods and techniques they employed. Gradient boosting is the most commonly used method, followed by random forest and regression (Fig. 2*A*). On average, gradient boosting appears more than once per submission, as each submission may include multiple software packages implementing different variants, such as XGBoost (27), CatBoost (28), and LightGBM (29). The LLMs’ frequent selection of gradient boosting aligns with its status as a top-performing method in recent machine learning challenges involving tabular and structured data (27). Neural networks, while widely used in fields such as imaging analysis, are rarely selected by LLMs, likely due to the lightweight and less complex nature of the datasets. Submissions that use gradient boosting achieve significantly better performance than those that do not (Fig. 2*B*), while submissions using random forest or regression tend to perform significantly worse. These findings reinforce the well-established effectiveness of gradient boosting in structured data tasks.

Most submissions involve only one type of machine learning method (e.g., gradient boosting), and nearly all include no more than four types (Fig. 2*C*), suggesting that LLMs tend to favor simpler modeling pipelines with a limited set of familiar algorithms rather than constructing complex ensembles. However, performance does not vary substantially with the number of machine learning methods used (Fig. 2*D*), indicating that increasing model diversity alone does not necessarily improve outcomes. Among machine learning techniques, parameter tuning is the most commonly used, appearing in nearly half of the submissions (Fig. 2*E*), reflecting LLMs’ recognition of the importance of hyperparameter optimization. Data imputation, feature engineering, and model averaging are also relatively common, showing awareness of standard preprocessing and ensembling strategies. In contrast, outlier removal is the least frequently used technique, suggesting either a lower emphasis on data cleaning or limitations in the prompt-driven setup regarding quality control. While most techniques are associated with improved performance, the gains are not statistically significant (Fig. 2*F*), implying that the value of these techniques may depend heavily on context and dataset characteristics.

### Prompt Strategies.

**Fig. 3.**
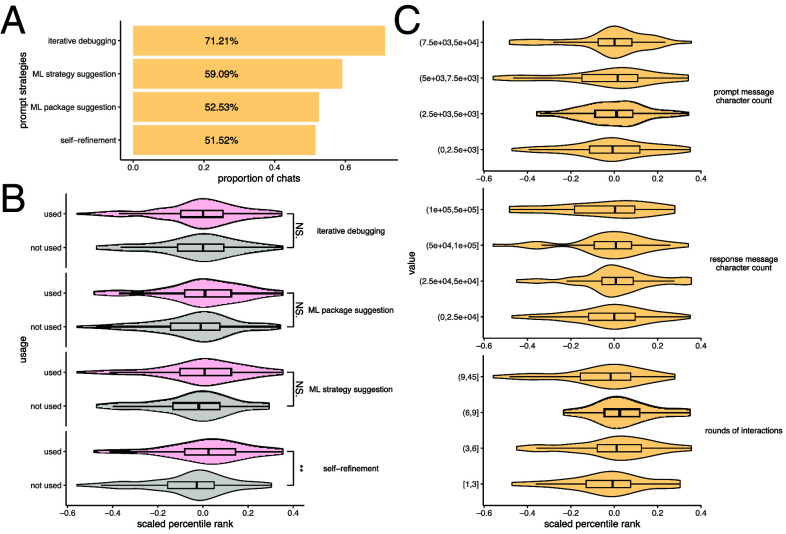
Prompt strategies. (*A*) Proportion of chats that used different prompt strategies. (*B*) Distribution of scaled percentile rank comparing solutions that did or did not use a given prompt strategy. Wilcoxon tests were performed to compare the distributions. “**” indicates a *P*-value between 0.001 and 0.01, and “NS.” indicates the *P*-value is not statistically significant. (*C*) Distribution of scaled percentile rank comparing chats across different intervals of character count in the prompt message, response message, and number of interaction rounds.

We next manually analyzed the prompt messages and curated the prompt strategies used by the students. Fig. 3*A* shows the proportion of cases in which different prompt strategies were employed. Here, a case refers to a complete chat history from one student addressing one Kaggle challenge. Iterative debugging, which involves repeatedly providing error messages to the LLMs and allowing them to fix errors in the code, is the most commonly used strategy. This suggests that while LLMs are prone to generating programming errors, they also possess a strong ability to correct their own mistakes, as indicated by the low percentage of final answers that failed to pass Kaggle’s evaluation system (Fig. 1*A*). In more than half of the cases, the name of a machine learning method or technique, such as those listed in Fig. 2, was mentioned in the chat. Self-refinement, a less commonly reported and studied prompt strategy, also appeared in about half of the chats. We define self-refinement as a prompt strategy in which the LLM critiques and revises its prior output without prescriptive guidance from the user, then returns an updated, drop in replacement solution aimed at improving a stated objective. For example, a user might first provide detailed instructions as an initial prompt, the LLM would produce an initial solution, and the user would then issue a follow up prompt such as “The performance is not good enough, further improve your answer,” which triggers the self-refinement step.

Fig. 3*B* compares the changes in performance when a specific prompt strategy is used versus not used. Only self-refinement substantially improves performance, while iterative debugging, ML package suggestion, and ML strategy suggestion do not lead to significant improvements. These results highlight the unique contribution of self-refinement to the effectiveness of LLM-assisted code generation. Unlike strategies that provide concrete feedback such as error messages or package suggestions, self-refinement prompts engage the LLMs in a more autonomous optimization process. This may encourage the model to explore a broader solution space or refine its reasoning, leading to more effective model choices or better hyperparameter tuning. The lack of significant improvement from other strategies suggests that simply providing error feedback or naming ML tools does not consistently lead to better outcomes, possibly because such information is already well-handled by the LLM without needing explicit instruction. Overall, these findings emphasize the value of prompting LLMs with open-ended, outcome-oriented instructions that allow them to self-direct their improvements.

We also found that other factors in a chat history, including the total number of characters in the prompt or response messages and the number of interaction rounds, have only a minor influence on performance (Fig. 3*C*). These findings suggest that longer or more interactive conversations with LLMs do not necessarily lead to better performance. This implies that the quality and type of prompt strategies, rather than the quantity of text or rounds of interaction, are more critical in influencing outcomes.

**Fig. 4.**
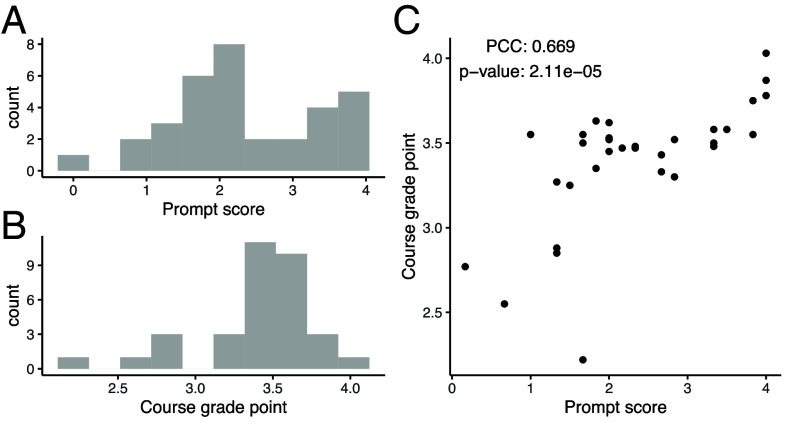
Prompt score and course grade point. (*A*) Distribution of prompt scores across students. (*B*) Distribution of course grade points across students. (*C*) Relationship between prompt scores and course grade points. Each point denotes one student. The Pearson correlation coefficient and *P*-value for the association between prompt scores and course grade points are shown in the *Upper Left*.

Based on these findings, we computed a prompt score for each student, defined as the average number of the four prompt strategies used across the six challenges, serving as a proxy for prompting skill (Fig. 4*A*). We also computed each student’s grade point by averaging performance across the six challenges according to the final project rubric (Fig. 4*B*). The prompt score correlated strongly with the grade point, with a Pearson correlation coefficient reaching 0.67, indicating that prompt skill is predictive of challenge performance, and that employing more prompt strategies generally yields higher scores.

**Fig. 5.**
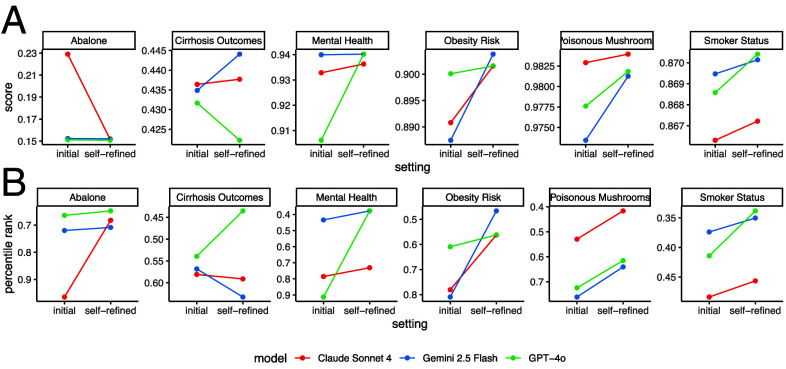
Validation of self-refinement. (*A* and *B*) Score (*A*) and percentile rank (*B*) comparing the initial and self-refined solutions.

Finally, we evaluated the effectiveness of the self-refinement strategy on three LLMs that were not used in the classroom experiment: GPT-4o, Gemini 2.5 Flash, and Claude Sonnet 4. For each of the six Kaggle data science challenges, each LLM was prompted to generate an initial solution and then produce a self-refined version (*Methods*). In nearly 90% of the cases, the self-refined solution outperformed the initial one and led to an average improvement of 11.5% in percentile rank (Fig. 5). These results align with the classroom experiment findings and suggest that the self-refinement strategy can be broadly applied to improve performance in data science challenges across different LLMs.

### More Complex Data Science Challenges.

**Fig. 6.**
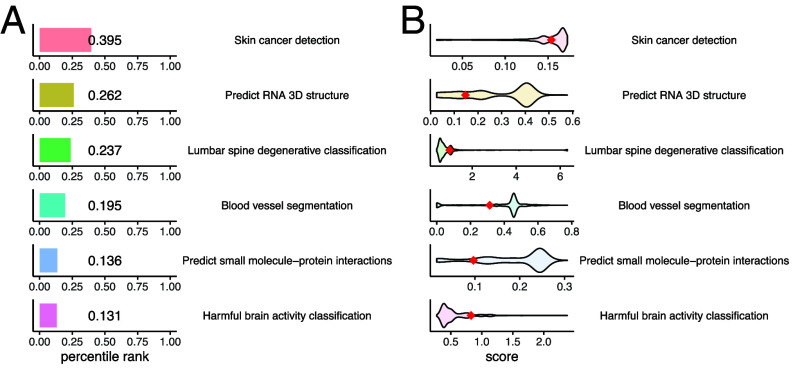
Performance of the o3 model on complex data science challenges. (*A*) Bar plots showing the percentile ranks of the o3 model across six additional Kaggle challenges involving more complex tasks. The numbers indicate the percentile ranks. (*B*) Violin plots displaying the distribution of scores from all human participants recorded on the Kaggle leaderboards for each challenge. The o3 model’s score is marked with red dots for comparison.

The six data science challenges used in the classroom experiment were all focused on tabular data prediction, which is relatively straightforward and does not require complex data processing or advanced modeling techniques. However, real-world data science problems are often more complex, involving multiple data modalities and requiring more sophisticated methods, such as deep learning. To test how well the OpenAI o3 model performs on these more difficult tasks, we evaluated it on six additional biomedical Kaggle challenges. These included segmenting blood vessels from HiP-CT images, detecting and classifying harmful brain activity using EEG signals, identifying histologically confirmed skin cancer from 3D total body photographs, diagnosing degenerative spine conditions from lumbar spine MRI scans, predicting the binding affinity of small molecules to protein targets, and inferring the 3D structure of RNA molecules from their sequences. For each task, we used a standardized prompt that included only the challenge description and dataset information from the Kaggle website, along with a self-refinement strategy. While the o3 model still outperformed about 20% of human participants (Fig. 6), its relative ranking was substantially lower compared to its performance on the classroom tabular challenges. Additionally, in several of these more advanced tasks, its performance score was substantially lower than that of the top human competitors.

The discrepancy in performance may be attributed to several factors. First, LLMs still face limitations in reasoning about complex tasks that involve intricate data structures or multiple modalities, such as images, signals, or 3D data. These tasks often require specialized preprocessing pipelines and model architectures beyond the scope of typical language-based reasoning. Second, many of these challenges benefit significantly from domain-specific knowledge, customized modeling approaches, and the use of external reference datasets. When prompted in a standardized manner, LLMs tend to default to generic machine learning strategies, which may be suboptimal for highly specialized biomedical problems. Third, unlike the tabular challenges used in the classroom experiment, which had no financial incentive and likely attracted less competitive participants, these more complex Kaggle challenges offered cash prizes and likely drew professional data scientists who invested considerable time and effort in crafting top-performing solutions. To address these limitations, future LLM development should focus on improving their ability to tackle complex, multimodal tasks by integrating domain-specific knowledge, tailoring modeling strategies to task context, and leveraging external data sources through retrieval- or tool-augmented frameworks.

## Discussions

We presented results from a classroom experiment in which graduate students used LLMs to participate in biomedical data science challenges hosted on Kaggle. We found that while students’ submissions did not top the leaderboards, their prediction scores were often close to those of top-performing human data scientists. LLMs tended to favor gradient boosting methods, and solutions incorporating these methods generally outperformed those that did not. Among various prompting strategies, the self-refinement approach, where the LLM is prompted to improve upon its own initial solution, proved to be the most effective. The effectiveness of this strategy was further validated using LLMs not included in the classroom setting. Overall, this study demonstrates that LLMs can reliably design machine learning algorithms with performance comparable to that of experienced human data scientists on relatively straightforward tasks, even when operated by users without specialized expertise in LLMs. However, more powerful LLMs equipped with domain-specific knowledge still need to be developed to effectively tackle more complex data science challenges. While this study does not focus on methodological innovations or new benchmark datasets, it provides a real-world evaluation of LLM performance in data science challenges and compares LLMs with human data scientists, offering valuable insights for practical deployment, human–AI collaboration, and the educational role of LLMs in data science and machine learning training.

Concepts related to self-refinement have appeared in recent work. One example is IMPROVE (30), an end-to-end LLM-agent framework that automates object classification pipelines through iterative, component-wise refinement. This approach aligns with expert practice by modifying one element at a time and linking each change to measurable validation feedback. In contrast to the human-in-the-loop refinement discussed in this study, IMPROVE provides a fully automated alternative that offers clear attribution of effective edits and follows a repeatable, metric-driven process. Despite its automation, human-in-the-loop refinement remains valuable for rapid prototyping and broad applicability. It can be executed directly within standard LLM interfaces without additional system setup, allows for fine-grained expert control, and can be especially useful when computational resources, data access, or environmental configurations are limited. These two strategies complement each other. Automation contributes rigor and consistency in evaluation, while human guidance brings flexibility, responsiveness, and domain-specific insight.

Online access for LLMs was disabled during the classroom experiment to prevent them from directly retrieving solutions submitted by human data scientists on challenge platforms, thereby ensuring a fairer evaluation setting. However, in real-world applications, LLMs are frequently used in conjunction with web browsing tools, plugins, and retrieval-augmented interfaces. To assess whether LLMs can benefit from such access, we conducted a follow-up experiment using the OpenAI o3 model to solve the same six data science challenges from the classroom study. The same prompt messages containing only the necessary information about the challenge and datasets were used, with and without online access enabled. We manually inspected the reasoning steps to confirm that the model did not directly retrieve solutions from the Kaggle website. While online access led to a modest improvement in overall performance, the difference was not statistically significant (*SI Appendix*, Fig. S1). While this study focuses on reporting the results of the classroom experiment, further research is warranted to explore how LLMs can more effectively leverage online resources to enhance their performance in data science tasks.

The classroom experiment design has several other limitations that should be considered when interpreting the results. First, it lacks a same classroom control group completing identical tasks without LLM assistance. Although we compared student performance to the cohort of human programmers participating in the same Kaggle challenges, these participants may differ from the students in programming experience, data science background, demographics, and domain knowledge. A within class control would enable a more direct comparison between LLM aided and unaided students, thereby strengthening causal interpretation of LLM utility. Implementing such a control is practically challenging, however, since splitting the cohort could compromise grading fairness and course equity, and it could create uneven workloads across groups. In addition, students assigned to a no LLM arm might still consult LLMs, and verifying compliance is technically challenging, thereby diminishing the credibility of the control condition. Second, this study was conducted within a single graduate-level course, involving students who share a relatively homogeneous academic background. While this design allowed for controlled observation of LLM usability in a focused educational setting, we recognize that such a sample may limit the generalizability of our conclusions. Future studies that replicate these experiments across multiple courses, disciplines, and learner populations, particularly those with varying levels of technical expertise, educational backgrounds, and professional experience, would be valuable for validating the reproducibility of our findings and establishing broader applicability in real-world learning environments. Third, students’ academic and demographic information was not collected or analyzed in this study due to ethical and privacy concerns. Including such information could provide additional context for interpreting the results of the classroom experiment. These limitations are worth addressing in future studies to more precisely characterize LLMs’ capabilities in solving data science challenges, and in code generation more broadly.

## Methods

### Kaggle Data Science Challenges.

The following six Kaggle data science challenges were involved in the classroom experiment:

1. Multi-Class Prediction of Obesity Risk (https://www.kaggle.com/competitions/playground-series-s4e2/).
2. Binary Prediction of Smoker Status using Bio-Signals (https://www.kaggle.com/competitions/playground-series-s3e24/).
3. Multi-Class Prediction of Cirrhosis Outcomes (https://www.kaggle.com/competitions/playground-series-s3e26/).
4. Binary Prediction of Poisonous Mushrooms (https://www.kaggle.com/competitions/playground-series-s4e8/).
5. Regression with an Abalone Dataset (https://www.kaggle.com/competitions/playground-series-s4e4).
6. Exploring Mental Health Data (https://www.kaggle.com/competitions/playground-series-s4e11/).

We analyzed the following six Kaggle challenges as representative examples of more complex data science tasks:

1. SenNet + HOA - Hacking the Human Vasculature in 3D (https://www.kaggle.com/competitions/blood-vessel-segmentation)
2. HMS - Harmful Brain Activity Classification (https://www.kaggle.com/competitions/hms-harmful-brain-activity-classification)
3. ISIC 2024 - Skin Cancer Detection with 3D-TBP (https://www.kaggle.com/competitions/isic-2024-challenge)
4. RSNA 2024 Lumbar Spine Degenerative Classification (https://www.kaggle.com/competitions/rsna-2024-lumbar-spine-degenerative-classification)
5. NeurIPS 2024 - Predict New Medicines with BELKA (https://www.kaggle.com/competitions/leash-BELKA)
6. Stanford RNA 3D Folding (https://www.kaggle.com/competitions/stanford-rna-3d-folding)

### Initial Instructions for the Final Project.

The following instructions for the final project were given to the students at the beginning of the course:

“You are required to participate in six Kaggle challenges. In the final project, you are NOT allowed to write your own code. Instead, you are required to provide prompts to OpenAI’s o1 model, which will generate the actual code.

For each task, you need to submit:

1. Your chat history URL with o1.
2. The code generated by o1. This should be the exact output from the o1 model and must be included in the chat history (which will be validated). This code will be used to evaluate performance. It is not required to be the last code generated by o1 in the entire chat history. It can be code generated earlier in the conversation. For instance, o1 may have generated some other code later, but you decided the previous code was better. The input can only be “train.csv” and “test.csv” provided by Kaggle.
3. A brief description of your prompt strategy and what you think contributed to the improvement of o1’s performance (you can provide one document for all six challenges).

When communicating with o1, you are only allowed to use natural language to instruct o1. Images are not permitted as input. You may recommend the name of the model, software package, or provide other useful information to o1, but only in natural language. Programming language is not restricted. You are prohibited from providing any actual programming code, or natural language directly translated from programming code (e.g., pseudocode) to o1. You will be notified and given a chance to fix it within a short timeframe if this happens. Violations will result in a score of 0 for the specific challenge and will disqualify you from the in-class ranking for the specific challenge and potential authorship of the paper.

For each challenge, you will receive a score of:

- 0 if any of the three required documents are missing, if the rules are violated, or if there is a bug in the final code that prevents obtaining a valid Kaggle ranking.
- 3.0 if you submit all three documents and the code is valid, resulting in a valid Kaggle ranking (regardless of percentile).
- 3.3 if you submit all three documents and your solution performs better than 30% of human participants on Kaggle.
- 3.5 if you submit all three documents and your solution performs better than 50% of human participants on Kaggle.
- 3.7 if you submit all three documents and your solution performs better than 70% of human participants on Kaggle.
- 4.0 if you submit all three documents and your solution performs better than 90% of human participants on Kaggle.

Bonus for each challenge:

- +1.0 if you rank first in class.
- +0.6 if you rank 2 to 3 in class.
- +0.3 if you rank 4 to 5 in class.

Each of the six challenges will be graded separately. The final score will be an average of the six scores for the six Kaggle challenges.”

### Updated Instructions for the Final Project.

The following instructions for the final project were given to the students after OpenAI replaced o1 with the o3 model:

“OpenAI has updated its model list, introducing the new o3 model while removing the older o1 model. Consequently, the rules for the final project have been revised.

You may complete the project with either the o1 or o3 model. Results from both models will be evaluated together, with no score adjustment to favor one over the other, so choosing o1 carries the risk that o3 may outperform it. If you opt for the o3 model, do not use the “Search the web,” “Deep research,” or “Canvas” functions. Usage of these features can be verified in your chat history. Projects that employ them will require resubmission.

All other project guidelines remain unchanged.

For those of you using o3, the model may still perform a web search even when the “Search the web” button is not toggled. In that case, disable web search in the settings: Click your user icon in the top right corner of the page, select “Customize ChatGPT,” scroll down to “Advanced,” and uncheck “Web search.” This will disable web search at the system level. Note: Changing the user settings is not required. If your response does not involve web browsing in the first place, please ignore this and you don’t need to redo anything.

Please double check that web browsing is not used by the o3 or o1 model in your response, as doing so violates the project rules. To verify, click “Thought for XX seconds” to view the step by step reasoning, and make sure no step shows “Searched the web” or something similar. If o3 or o1 performs web browsing in your submission, we will return it and ask you to redo it.”

### Evaluation of Self-Refinement Strategy.

We prompted the online versions of GPT-4o, Gemini 2.5 Flash, and Claude Sonnet 4 to generate programming code for solving the same six Kaggle data science challenges used in the classroom experiment. For the initial query, the prompt message consisted of the following components:

- “Write code for performing the following machine learning challenge:”
- Information about the challenge copied from the Kaggle website.
- The first 10 lines of the training file, test file, and example submission file.
- “Generate code in a single chunk. Do not provide explanations. The code cannot take longer than 1 h to finish.”

For the self-refinement query, the prompt message is as follows: “The performance is not good enough. Further improve your answer.”

### Calculation of Scaled Percentile Rank.

We introduced the scaled percentile rank to account for differences in percentile rank distributions across Kaggle challenges, enabling pooled analysis across challenges. For each Kaggle data science challenge, the scaled percentile rank was calculated by subtracting the median percentile rank of all students in that challenge from each student’s raw percentile rank.

### Ethics Statement.

This study was reviewed by the Duke Health Institutional Review Board (DUHS IRB) and determined not to involve human subjects (Protocol Pro00117372).

## Supplementary Material

Appendix 01 (PDF)

## Data Availability

All study data are included in the article and/or *SI Appendix*.
