# Supplementary material for: Evaluating large language models in biomedical data science challenges through a classroom experiment
Source: Proc Natl Acad Sci U S A. 2025 Dec 11;122(50):e2521062122. doi: 10.1073/pnas.2521062122 (PMC12718336; doi:10.1073/pnas.2521062122)
Supplement: Supplementary file 1 — Appendix 01 (PDF) [file pnas.2521062122.sapp.pdf]

Supplementary materials

Supplementary Table 1. This table contains the deidentified performance data of students across the six data science challenges in the classroom experiment. The columns are organized as follows: deidentified student ID, task name, programming language used, LLM model used, Kaggle private score, Kaggle ranking, scaled Kaggle ranking, machine learning models and techniques mentioned in the response message, number of conversation rounds with the LLM, proportion of errors in the response message, number of characters in the prompt and response messages, and prompt strategies used in the prompt message.

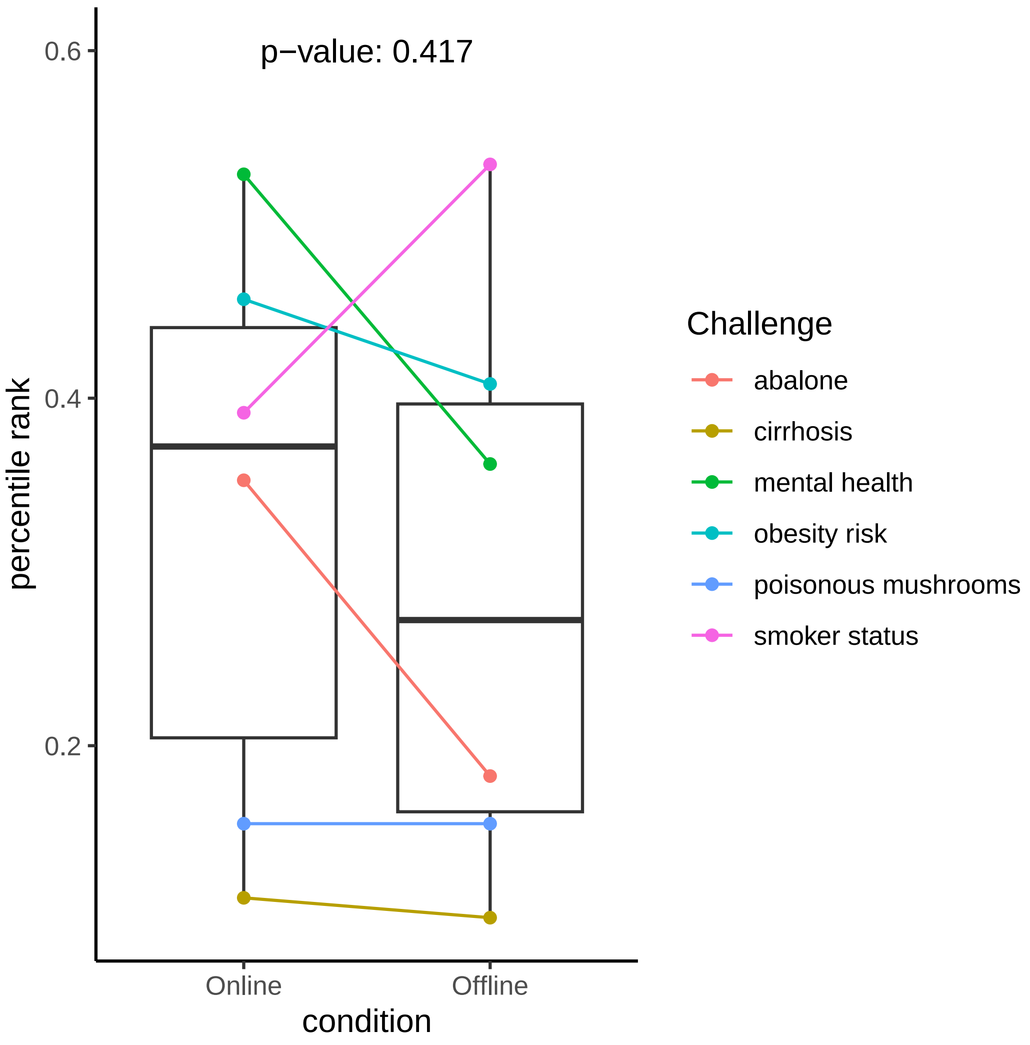

Supplementary Figure 1. Comparison of the o3 model's percentile ranks with and without online access, shown on the y-axis and x-axis, respectively. Data points and connecting lines are color-coded by the six data science challenges from the classroom experiment. The p-value was calculated using a two-sided paired two-sample t-test.

student_in	task_index	programmi	LLM_mode	kaggle_priv	kaggle_ran	scaled_kag	MLstrategy	MLstrategy
1	obesity risk	R	o3	0.90101	0.421566	-0.18167	randomFor	Y
1	smoker sta	R	o3	0.86925	0.616754	-0.0199	glmnet; rar	Y
1	cirrhosis	R	o3	0.41214	0.684907	0.112447	xgboost	Y
1	poisonous	R	o1	0.98306	0.485149	-0.00784	ranger	N
1	abalone	R	o3	0.14732	0.674463	0.246933	xgboost	Y
1	mental hea	R	o3	0.93899	0.428731	-0.16189	xgboost; ra	N
2	obesity risk	Python	o3	0.90489	0.603232	0	xgboost;ra	Y
2	smoker sta	Python	o3	0.86939	0.62199	-0.01466	xgboost;ra	Y
2	cirrhosis	Python	o3	0.42098	0.583283	0.010824	xgboost;ra	Y
2	poisonous	Python	o3	0.67199	0.134901	-0.35809	elastic-net	N
2	abalone	Python	o3	0.1519	0.297929	-0.1296	xgboost;ra	Y
2	mental hea	Python	o3	0.93825	0.36323	-0.22739	xgboost;ra	Y
3	obesity risk	R	o3	0.90652	0.728615	0.125383	xgboost	N
3	smoker sta	R	o1	0.87172	0.721466	0.084817	xgboost	N
3	cirrhosis	R	o1	0.42067	0.585087	0.012628	xgboost	N
3	poisonous	R	o1	0.98358	0.542492	0.049505	lightgbm	N
3	abalone	R	o1	0.14932	0.427531	0	xgboost	Y
3	mental hea	R	o1	0.9384	0.379606	-0.21102	ranger	N
4	obesity risk	Python	o3	0.89315	0.256617	-0.34661	LogisticReg	N
4	smoker sta	Python	o3	0.8599	0.399476	-0.23717	GradientBo	N
4	cirrhosis	Python	o3	NA	NA	NA	LogisticReg	N
4	poisonous	Python	o3	0.638	0.130776	-0.36221	LogisticReg	N
4	abalone	Python	o3	0.15906	0.16296	-0.26457	LinearRegr	N
4	mental hea	Python	o3	NA	NA	NA	LogisticReg	N
5	obesity risk	R	o1	0.90182	0.444692	-0.15854	randomFor	Y
5	smoker sta	R	o1	0.86347	0.449215	-0.18743	xgboost	Y
5	cirrhosis	R	o1	0.42019	0.5911	0.018641	xgboost; ra	Y
5	poisonous	R	o1	0.98474	0.75165	0.258663	randomFor	N
5	abalone	R	o1	0.15028	0.37615	-0.05138	xgboost	Y
5	mental hea	R	o1	NA	NA	NA	randomFor	Y
6	obesity risk	R	o3	0.90074	0.413207	-0.19003	xgboost	N
6	smoker sta	R	o3	0.86975	0.636649	0	xgboost	N
6	cirrhosis	R	o3	0.56274	0.122069	-0.45039	xgboost	N
6	poisonous	R	o3	NA	NA	NA	xgboost; ra	N
6	abalone	R	o3	0.15076	0.353144	-0.07439	xgboost	N
6	mental hea	R	o3	0.94083	0.870115	0.279494	xgboost	N
7	obesity risk	Python	o3	0.89315	0.256617	-0.34661	GradientBo	N
7	smoker sta	Python	o3	0.86969	0.634555	-0.00209	LightGBM	N
7	cirrhosis	Python	o3	0.43068	0.464221	-0.10824	catboost	N
7	poisonous	Python	o3	0.98363	0.544967	0.05198	catboost	N
7	abalone	Python	o3	0.14965	0.409126	-0.0184	GradientBo	N
7	mental hea	Python	o3	NA	NA	NA	lightgbm	N
8	obesity risk	Python	o1	0.90444	0.571468	-0.03176	xgboost;lig	N
8	smoker sta	Python	o1	0.86848	0.582723	-0.05393	lightgbm	Y
8	cirrhosis	Python	o1	0.42388	0.544799	-0.02766	xgboost	Y
8	poisonous	Python	o1	0.98417	0.631601	0.138614	xgboost	Y

8	abalone	Python	o3	0.15067	0.358129	-0.0694	lightgbm	Y
8	mental hea	Python	o3	0.93969	0.512467	-0.07815	lightgbm	Y
9	obesity risk	Python	o1	0.90724	0.787127	0.183895	xgboost	Y
9	smoker sta	Python	o1	0.87177	0.725654	0.089005	catboost	Y
9	cirrhosis	Python	o1	0.41381	0.671076	0.098617	xgboost	Y
9	poisonous	Python	o1	0.98321	0.500825	0.007838	catboost	N
9	abalone	Python	o1	0.14659	0.769555	0.342025	lightgbm;ca	N
9	mental hea	Python	o1	0.94101	0.919613	0.328991	lightgbm;ca	N
10	obesity risk	Python	o3	0.9048	0.598495	-0.00474	xgboost	Y
10	smoker sta	Python	o3	0.87179	0.726702	0.090052	xgboost	Y
10	cirrhosis	Python	o3	0.40765	0.742634	0.170174	xgboost	Y
10	poisonous	Python	o3	0.9846	0.717409	0.224422	xgboost	Y
10	abalone	Python	o3	0.15143	0.321702	-0.10583	xgboost;ca	Y
10	mental hea	Python	o3	0.94023	0.63342	0.042799	catboost	Y
11	obesity risk	Python	o3	0.89342	0.262747	-0.34048	lightgbm	N
11	smoker sta	Python	o3	0.86319	0.444503	-0.19215	lightgbm	Y
11	cirrhosis	Python	o3	0.44287	0.375827	-0.19663	catboost	N
11	poisonous	Python	o3	0.98327	0.509076	0.016089	catboost	N
11	abalone	Python	o3	0.14717	0.697853	0.270322	xgboost	N
11	mental hea	Python	o3	0.93121	0.196874	-0.39375	lightgbm	N
12	obesity risk	Python	o3	0.90606	0.678741	0.075508	lightgbm	N
12	smoker sta	Python	o3	0.86835	0.579581	-0.05707	lightgbm	Y
12	cirrhosis	Python	o3	0.41786	0.622369	0.04991	lightgbm	Y
12	poisonous	Python	o3	0.98421	0.639439	0.146452	lightgbm	N
12	abalone	Python	o3	0.15019	0.380752	-0.04678	lightgbm;ca	N
12	mental hea	Python	o3	0.94077	0.858951	0.268329	lightgbm;ca	N
13	obesity risk	Python	o3	0.90625	0.690443	0.087211	xgboost;lig	Y
13	smoker sta	Python	o3	0.87149	0.709948	0.073298	xgboost;lig	Y
13	cirrhosis	Python	o3	0.42083	0.583885	0.011425	xgboost;lig	Y
13	poisonous	Python	o1	0.98435	0.662129	0.169142	randomFor	N
13	abalone	Python	o3	0.14738	0.666794	0.239264	xgboost;lig	Y
13	mental hea	Python	o3	0.94061	0.805359	0.214738	xgboost;lig	Y
14	obesity risk	R	o1	0.90101	0.421566	-0.18167	randomFor	N
14	smoker sta	R	o1	0.78445	0.178534	-0.45812	randomFor	N
14	cirrhosis	R	o3	0.42442	0.538785	-0.03367	xgboost	N
14	poisonous	R	o3	0.98324	0.505776	0.012789	xgboost	N
14	abalone	R	o3	0.14657	0.771472	0.343942	xgboost; ra	Y
14	mental hea	R	o1	0.81687	0.032378	-0.55824	ranger	N
15	obesity risk	Python	o3	0.90643	0.721649	0.118417	xgboost; ra	Y
15	smoker sta	Python	o3	0.87034	0.657068	0.020419	LogisticReg	Y
15	cirrhosis	Python	o3	0.6369	0.101022	-0.47144	XGBoost	N
15	poisonous	Python	o3	0.98286	0.465347	-0.02764	LightGBM	N
15	abalone	Python	o3	0.15194	0.294479	-0.13305	GradientBo	Y
15	mental hea	Python	o3	0.93582	0.255675	-0.33495	RandomFo	N
16	obesity risk	Python	o3	0.90281	0.489273	-0.11396	catboost	Y
16	smoker sta	Python	o3	0.86985	0.639791	0.003141	LogisticReg	Y
16	cirrhosis	Python	o3	0.4167	0.634396	0.061936	xgboost	Y

16	poisonous	Python	o3	0.90326	0.161304	-0.33168	GradientBo	N
16	abalone	Python	o3	0.15066	0.358896	-0.06863	RandomFo	Y
16	mental hea	Python	o3	0.94032	0.66617	0.075549	xgboost	Y
17	obesity risk	Python	o1	0.90652	0.728615	0.125383	xgboost	Y
17	smoker sta	Python	o1	0.8687	0.593717	-0.04293	xgboost	Y
17	cirrhosis	Python	o1	0.42603	0.521347	-0.05111	xgboost	Y
17	poisonous	Python	o1	0.97656	0.262376	-0.23061	xgboost	Y
17	abalone	Python	o1	0.15079	0.352761	-0.07477	xgboost	Y
17	mental hea	Python	o1	0.94061	0.805359	0.214738	xgboost	Y
18	obesity risk	Python	o1	0.90534	0.635274	0.032042	RandomFo	N
18	smoker sta	Python	o1	0.87028	0.65445	0.017801	xgboost;lig	N
18	cirrhosis	Python	o1	0.42797	0.494889	-0.07757	xgboost;lig	N
18	poisonous	Python	o1	NA	NA	NA	xgboost;lig	N
18	abalone	Python	o1	0.14641	0.782975	0.355445	catboost	N
18	mental hea	Python	o1	0.93999	0.577596	-0.01303	xgboost;lig	N
19	obesity risk	R	o3	0.90615	0.683756	0.080524	lightgbm	Y
19	smoker sta	R	o3	0.87066	0.679058	0.042408	xgboost	Y
19	cirrhosis	R	o3	0.41556	0.648827	0.076368	xgboost;lig	N
19	poisonous	R	o3	0.98422	0.641502	0.148515	lightgbm	N
19	abalone	R	o3	0.14741	0.663727	0.236196	lightgbm;xg	N
19	mental hea	R	o3	0.94012	0.605136	0.014514	xgboost;lig	Y
20	obesity risk	R	o1	0.90498	0.610755	0.007523	randomFor	Y
20	smoker sta	R	o1	0.86478	0.47801	-0.15864	logistic reg	Y
20	cirrhosis	Python	o1	0.41888	0.614552	0.042093	xgboost	Y
20	poisonous	R	o1	0.98441	0.673267	0.180281	ranger	Y
20	abalone	Python	o1	0.15079	0.352761	-0.07477	xgboost	Y
20	mental hea	Python	o1	0.94007	0.590622	0	xgboost	Y
21	obesity risk	R	o1	0.90634	0.696573	0.093341	xgboost	N
21	smoker sta	R	o1	0.78843	0.22356	-0.41309	xgboost	N
21	cirrhosis	R	o1	0.42231	0.564642	-0.00782	xgboost	N
21	poisonous	Python	o1	0.98228	0.401815	-0.09117	lightgbm;ca	N
21	abalone	Python	o1	NA	NA	NA	catboost	N
21	mental hea	Python	o1	NA	NA	NA	lightgbm	N
22	obesity risk	Python	o3	0.90724	0.787127	0.183895	xgboost;lig	N
22	smoker sta	Python	o1	0.87086	0.687435	0.050785	lightgbm;xg	Y
22	cirrhosis	Python	o3	0.4064	0.760072	0.187613	lightgbm;xg	Y
22	poisonous	Python	o1	0.98441	0.673267	0.180281	DecisionTre	Y
22	abalone	Python	o3	NA	NA	NA	TabularPre	N
22	mental hea	Python	o3	0.91533	0.107555	-0.48307	LogisticReg	Y
23	obesity risk	Python	o1	0.90408	0.549178	-0.05405	lightgbm	N
23	smoker sta	Python	o3	0.8691	0.609948	-0.0267	lightgbm	N
23	cirrhosis	Python	o1	0.42414	0.540589	-0.03187	lightgbm	N
23	poisonous	Python	o3	0.98065	0.335809	-0.15718	lightgbm	N
23	abalone	Python	o1	0.14758	0.637653	0.210123	lightgbm	N
23	mental hea	Python	o3	0.93997	0.574246	-0.01638	lightgbm	N
24	obesity risk	R	o3	0.90661	0.736138	0.132906	xgboost	N
24	smoker sta	R	o3	0.8716	0.715183	0.078534	xgboost	N

24	cirrhosis	R	o3	0.48677	0.239928	-0.33253	xgboost	Y
24	poisonous	R	o3	0.97747	0.273102	-0.21988	lightgbm	N
24	abalone	R	o3	0.24572	0.030675	-0.39686	glmnet; xgb	N
24	mental hea	R	o3	0.93905	0.43543	-0.15519	lightgbm	N
25	obesity risk	R	o1	0.9039	0.537197	-0.06604	xgboost	Y
25	smoker sta	R	o1	0.86746	0.553403	-0.08325	lightgbm	N
25	cirrhosis	R	o1	0.42301	0.55923	-0.01323	xgboost	N
25	poisonous	R	o1	0.98331	0.512376	0.019389	catboost	N
25	abalone	R	o1	0.1504	0.373083	-0.05445	catboost	N
25	mental hea	R	o1	0.94087	0.880536	0.289914	catboost	N
26	obesity risk	R	o3	0.90317	0.503761	-0.09947	xgboost	Y
26	smoker sta	R	o3	0.86182	0.426702	-0.20995	ranger	N
26	cirrhosis	R	o3	0.42023	0.5911	0.018641	xgboost	Y
26	poisonous	R	o3	NA	NA	NA	ranger	N
26	abalone	R	o3	0.14699	0.721626	0.294095	xgboost	N
26	mental hea	R	o3	0.93556	0.248232	-0.34239	glmnet	N
27	obesity risk	Python	o1	0.90299	0.495124	-0.10811	lightgbm	Y
27	smoker sta	Python	o1	0.87169	0.720942	0.084293	lightgbm	Y
27	cirrhosis	Python	o3	0.42271	0.56344	-0.00902	xgboost	Y
27	poisonous	Python	o3	0.98115	0.353135	-0.13985	lightgbm	N
27	abalone	Python	o3	0.14695	0.731212	0.303681	lightgbm	Y
27	mental hea	Python	o3	0.94072	0.844808	0.254187	lightgbm	Y
28	obesity risk	Python	o3	0.90417	0.553915	-0.04932	lightgbm	N
28	smoker sta	Python	o3	0.87206	0.736649	0.1	xgboost	N
28	cirrhosis	Python	o3	0.60654	0.106434	-0.46603	catboost	N
28	poisonous	Python	o3	0.98088	0.34571	-0.14728	lightgbm	N
28	abalone	Python	o3	0.1489	0.461656	0.034126	HistGradien	N
28	mental hea	Python	o3	0.94008	0.595087	0.004466	catboost	N
29	obesity risk	Python	o1	0.90399	0.541655	-0.06158	LogisticReg	Y
29	smoker sta	Python	o1	0.87173	0.72199	0.08534	RandomFo	N
29	cirrhosis	Python	o1	0.4412	0.387252	-0.18521	RandomFo	N
29	poisonous	Python	o1	0.98235	0.408003	-0.08498	xgboost	Y
29	abalone	Python	o1	0.14805	0.552914	0.125383	catboost;xg	N
29	mental hea	Python	o1	0.93955	0.489393	-0.10123	lightgbm;Lo	Y
30	obesity risk	Python	o3	0.90606	0.678741	0.075508	LogisticReg	N
30	smoker sta	Python	o3	0.86961	0.631937	-0.00471	lightgbm	Y
30	cirrhosis	Python	o3	0.42135	0.580277	0.007817	xgboost	Y
30	poisonous	Python	o3	0.9643	0.212046	-0.28094	lightgbm	Y
30	abalone	Python	o3	0.14711	0.705138	0.277607	lightgbm	N
30	mental hea	Python	o3	0.94056	0.761816	0.171195	lightgbm	N
31	obesity risk	R	o1	0.90679	0.749234	0.146002	xgboost	Y
31	smoker sta	R	o1	0.87156	0.713089	0.07644	xgboost;lig	N
31	cirrhosis	R	o1	0.42739	0.499699	-0.07276	xgboost	Y
31	poisonous	R	o1	0.53457	0.12335	-0.36964	ranger	N
31	abalone	R	o1	0.15618	0.193252	-0.23428	randomFor	N
31	mental hea	R	o3	0.93996	0.568664	-0.02196	glmnet;xgb	Y
32	obesity risk	R	o3	0.90606	0.678741	0.075508	xgboost	Y

32	smoker sta	R	o3	0.87192	0.732461	0.095812	xgboost	N
32	cirrhosis	R	o3	0.41642	0.638004	0.065544	xgboost	N
32	poisonous	R	o3	0.98422	0.641502	0.148515	xgboost	Y
32	abalone	R	o3	0.14896	0.457822	0.030291	xgboost	Y
32	mental hea	R	o3	0.94049	0.741347	0.150726	lightgbm;gl	Y
33	obesity risk	Python	o3	0.9067	0.743104	0.139872	xgboost	Y
33	smoker sta	Python	o3	0.87054	0.675393	0.038743	xgboost	Y
33	cirrhosis	Python	o3	0.42004	0.592303	0.019844	xgboost	Y
33	poisonous	Python	o3	0.98254	0.421205	-0.07178	catboost	Y
33	abalone	Python	o3	0.14805	0.552914	0.125383	xgboost	Y
33	mental hea	Python	o3	0.94108	0.940826	0.350205	xgboost	Y

MLstrategy	MLstrategy	MLstrategy	MLstrategy	MLstrategy	prompt_nu	prompt_er	prompt_nc	response_r
8	N	N	N	Y	17	0.411765	3673	130681
16	Y	N	Y	Y	45	0.377778	8149	336560
120	N	N	N	Y	1	0	1382	4913
NA	N	N	Y	N	17	0.294118	9484	127679
100	N	N	Y	Y	11	0.181818	2396	52640
NA	N	N	Y	Y	25	0.8	3987	139032
120	N	N	N	N	2	1	2141	17213
75	N	N	N	N	2	0.5	1987	8218
40	N	N	Y	N	4	0.5	1910	26197
NA	N	N	Y	N	6	0.5	2878	42150
150	N	N	N	N	2	0.5	1954	16479
69	N	N	Y	N	2	1	2105	20081
NA	N	N	Y	N	1	0	909	6280
NA	N	N	Y	N	4	0.75	3704	20734
NA	N	N	N	N	3	1	2679	12672
NA	N	N	N	N	8	0.5	6262	27815
12	N	N	Y	N	1	1	969	6031
NA	N	N	Y	N	3	0.333333	2343	12586
NA	N	N	N	N	1	0	351	1049
NA	N	Y	N	N	1	0	1826	4840
NA	N	N	Y	N	1	0	1347	5913
NA	N	N	N	N	2	0	473	5062
NA	Y	N	N	N	1	0	370	671
NA	N	N	N	N	1	0	311	1040
4	N	Y	N	N	26	0.423077	42901	149550
32	N	Y	Y	N	20	0.25	5599	134715
8	N	N	N	N	8	0.125	6775	55355
NA	N	N	Y	N	9	0.444444	4251	59172
648	N	N	N	N	8	0.25	2769	33176
10	N	N	N	N	17	0.411765	9306	83217
NA	N	N	N	N	1	0	2925	3509
NA	N	N	N	N	2	0.5	4023	7164
NA	N	N	N	N	6	0.166667	6025	26096
NA	N	N	N	Y	6	0.5	4437	29206
NA	N	N	N	N	3	0.666667	3054	9670
NA	N	N	N	N	23	0.478261	6603	100599
NA	N	N	N	N	2	0	862	6175
NA	N	Y	N	N	6	0.5	1551	19719
NA	N	N	N	N	2	0	1439	8154
NA	N	N	Y	N	2	0	1385	6081
NA	N	N	Y	N	5	0	1384	11854
NA	N	N	Y	N	4	0	5609	10340
NA	N	N	N	Y	11	0.272727	5355	57564
20	N	N	N	N	3	0.333333	1182	13154
64	N	N	N	N	9	0.444444	8577	43045
20	N	N	N	N	5	0.2	1811	24703

30	N	Y	N	N		10	0.3	3648	71003
50	N	N	Y	N		4	0	1150	11764
30	N	N	N	N		4	0.5	6326	24256
12	N	Y	N	N		3	0.333333	4123	15495
150	N	N	N	N		9	0.666667	28334	64743
NA	N	Y	N	N		6	0.5	11198	28210
NA	N	N	N	N		6	0.5	6903	50017
NA	N	N	N	Y		4	0.5	7220	36421
150	N	Y	Y	N		12	0.333333	12095	65317
100	N	Y	Y	N		8	0.75	12565	47112
100	N	Y	Y	N		5	0.4	8338	44503
120	N	Y	Y	N		5	0.6	9804	21967
400	N	Y	Y	N		12	0.333333	11569	89308
200	N	Y	Y	N		8	0.25	7985	49115
NA	N	N	N	N		5	0.4	6512	13063
15	N	Y	N	N		4	0	2472	16743
NA	N	N	N	N		6	0.333333	9341	19465
NA	N	N	N	N		6	0	1889	25153
NA	N	Y	Y	N		2	0.5	1752	9345
NA	N	N	Y	N		4	0.25	2208	12253
NA	N	Y	N	N		3	0.666667	3551	15503
12	N	Y	N	N		13	0.461538	9933	46178
25	N	N	N	N		4	0.5	7386	24552
NA	N	N	N	N		4	0.25	5172	18402
NA	N	Y	N	Y		13	0.307692	8259	65240
NA	N	N	Y	Y		3	0.333333	7134	15525
54	N	Y	Y	Y		6	0	2508	30411
189	N	N	Y	Y		1	0	1343	6536
54	N	Y	Y	Y		4	0.25	2900	24177
NA	N	Y	Y	N		7	0	1955	32225
162	N	N	N	Y		5	0.2	2168	26872
243	N	N	Y	N		3	0	1962	17837
NA	N	N	N	N		8	0.25	2371	42653
NA	N	Y	N	Y		27	0.259259	13118	223993
NA	N	N	N	N		5	0.4	1964	23691
NA	N	N	Y	N		19	0.315789	5963	91627
96	Y	Y	N	N		8	0.375	4380	35437
NA	N	N	Y	N		15	0.333333	5206	97479
80	N	Y	Y	N		5	0	2117	41399
160	N	Y	Y	Y		2	0	1630	18345
NA	N	Y	Y	N		1	0	1645	6737
NA	N	Y	Y	N		1	0	1170	7560
12	N	Y	Y	N		1	1	1534	10366
NA	N	Y	Y	Y		5	0.2	3151	53030
40	N	N	N	N		2	0	1252	10836
80	N	Y	Y	N		2	0	1391	11395
250	N	Y	Y	N		4	0.25	1890	28932

NA	N	N	N	N	4	0	1481	9113
40	N	Y	N	N	3	0.333333	1636	22795
40	N	Y	Y	N	1	0	1019	5609
20	N	N	N	N	2	0	2971	11859
96	N	N	N	N	6	0	4306	32989
108	N	N	Y	N	3	0	5888	21526
36	N	Y	Y	N	1	0	4223	6834
100	N	N	N	N	2	1	4799	11075
144	N	Y	Y	N	2	0	3939	12798
NA	N	Y	N	Y	15	0.133333	16502	123185
NA	N	Y	N	Y	9	0.444444	13051	59519
NA	N	Y	N	Y	6	0.5	8272	41005
NA	N	N	N	N	7	0.714286	19861	6737
NA	N	Y	N	N	5	0.6	12581	25474
NA	N	N	Y	Y	16	0.875	19039	45597
32	N	N	N	N	11	0.090909	4493	72463
36	N	N	N	N	34	0.088235	6762	166769
NA	N	Y	N	N	16	0.1875	5998	46025
NA	N	N	N	Y	25	0.16	8222	114442
NA	N	Y	N	Y	39	0.358974	18159	232748
4	Y	N	N	Y	35	0.4	8547	215798
58	N	Y	N	N	8	0.125	15261	47166
14	N	Y	N	N	9	0	2627	41228
128	N	N	Y	N	4	0.25	2953	18499
12	N	N	Y	N	8	0	2500	30329
216	N	N	N	N	2	0.5	1895	11186
16	N	N	Y	N	1	0	2190	7737
NA	N	N	N	N	4	0	2844	9240
NA	N	N	N	N	32	0.59375	6292	128539
NA	N	N	Y	N	1	0	2109	5520
NA	N	N	N	Y	7	0.428571	2517	24389
NA	N	N	Y	N	5	0.6	3187	18348
NA	N	N	N	N	7	0.428571	6221	24296
NA	N	Y	N	Y	5	0	4797	20682
60	N	Y	Y	Y	24	0.333333	26088	244862
81	N	Y	Y	Y	17	0.588235	9223	65959
1080	N	N	N	N	30	0.366667	23739	237833
NA	N	Y	N	N	15	0.666667	6842	40557
15	N	Y	Y	N	36	0.194444	12467	105828
NA	N	N	N	N	9	0	1135	36999
NA	Y	N	N	N	8	0.125	1369	26809
NA	Y	N	N	N	2	0	1366	8716
NA	N	N	N	N	5	0	1994	21824
NA	Y	N	N	N	3	0.333333	1275	14087
NA	Y	N	N	N	8	0	2258	45357
NA	N	N	N	N	24	0.458333	5899	78055
NA	N	Y	Y	N	19	0.368421	17107	54077

100	N	Y	N	N	19	0.368421	14383	59941
NA	N	N	N	N	11	0.363636	7806	44164
NA	N	Y	Y	Y	19	0.263158	8488	62682
NA	N	N	Y	N	14	0.142857	5743	53802
12	N	N	N	N	4	0.25	3407	22588
NA	N	N	Y	N	10	0.2	2677	41935
NA	N	Y	N	N	13	0.307692	6427	55464
NA	N	N	N	N	6	0.5	4119	21080
NA	N	Y	N	N	12	0.583333	7424	52378
NA	N	N	N	N	2	0.5	2342	7533
8	N	Y	N	N	20	0.4	6811	74644
NA	N	N	N	N	4	0.5	3328	13524
2	N	Y	Y	N	17	0.176471	4962	73599
NA	N	N	N	N	5	0.4	3062	14059
NA	N	Y	N	N	2	0.5	2131	3622
NA	N	N	Y	N	14	0.714286	4303	48502
16	N	N	N	N	3	0	2098	15744
50	N	N	Y	N	6	0.166667	5781	42799
40	N	N	Y	N	5	0.4	3174	24676
NA	N	N	Y	N	5	0.2	3243	24510
50	N	N	N	N	2	0.5	1772	10851
10	N	N	Y	N	4	0.25	3435	22353
NA	N	Y	N	N	5	0.6	12508	24238
NA	N	N	N	N	3	0.333333	7006	7568
NA	N	N	N	N	2	0	6217	9466
NA	N	N	N	N	3	0.333333	7411	5543
NA	N	N	N	N	2	0.5	6158	7717
NA	N	N	N	N	2	0	7359	6972
36	N	N	N	N	7	0	3525	59176
NA	N	N	N	Y	5	0.2	2993	30852
NA	N	Y	Y	N	4	0.25	2575	28352
6	N	Y	Y	N	13	0.230769	4837	87163
NA	N	Y	N	Y	4	1	1499	23295
10	N	Y	Y	Y	14	0.285714	3505	79178
NA	N	N	N	N	11	0.181818	10549	64368
40	N	N	N	N	5	0.4	9225	30836
192	N	N	N	N	13	0.538462	40271	97742
40	N	N	Y	N	20	0.4	22954	184390
NA	N	Y	N	Y	7	0.571429	4740	36257
NA	N	N	Y	N	11	0.727273	20811	68592
20	N	Y	N	N	5	0.2	8291	16091
NA	N	N	Y	Y	31	0.516129	17628	190736
8	N	N	N	N	7	0	15502	34323
NA	N	N	Y	N	15	0.733333	19562	60578
NA	N	Y	N	Y	7	0.142857	5611	23261
8	N	N	Y	N	13	0.384615	12343	47876
432	N	Y	N	N	12	0.25	4181	50834

NA	N	Y	N	N		9	0.222222	4103	24858
NA	N	N	N	N		13	0	4931	57867
9	N	N	Y	N		18	0.444444	6543	87240
30	N	Y	N	N		7	0.142857	2997	20354
50	N	N	Y	Y		20	0.3	4303	105107
25	N	N	Y	N		8	0.125	2489	14210
50	N	Y	N	N		7	0	1742	10358
40	N	Y	N	N		7	0	2675	10745
8	N	N	N	N		13	0.307692	3796	17579
27	N	N	N	N		8	0.125	2533	12199
40	N	N	Y	N		9	0.111111	2436	12384

promptstra	promptstra	promptstra	promptstrategy_selfrefine
1	1	1	1
1	1	1	1
0	1	0	0
1	1	1	1
0	1	1	1
1	1	1	1
1	1	1	0
1	1	0	0
1	1	1	0
1	1	1	0
1	1	1	0
1	1	1	0
0	1	0	0
0	1	1	0
0	1	1	0
0	1	1	0
0	1	0	0
0	1	1	0
0	1	0	0
0	0	0	0
0	1	0	0
0	0	0	0
0	1	0	0
0	1	0	0
0	0	1	1
0	0	1	1
0	0	1	1
0	0	1	1
0	0	1	1
0	0	1	1
0	0	0	0
0	0	1	0
1	1	1	0
0	0	1	0
0	0	1	0
0	0	1	1
0	0	0	0
0	0	1	0
0	0	0	0
0	0	0	0
0	0	0	0
0	0	0	0
1	1	1	0
0	0	1	1
1	1	1	0
1	0	0	1

1	1	1	0
0	0	0	0
1	1	1	1
1	1	1	1
1	1	1	1
1	1	1	1
1	1	1	1
1	1	1	1
1	1	1	1
1	1	1	1
1	1	1	1
1	1	1	1
1	1	1	1
1	1	1	1
1	1	1	0
0	0	1	0
1	1	1	0
0	0	0	0
0	0	0	0
0	0	1	0
0	0	1	0
0	0	1	1
0	0	1	0
0	0	1	1
0	0	1	1
0	0	1	1
1	1	0	0
0	0	0	0
1	1	1	0
1	1	0	1
1	1	0	0
1	1	0	0
1	0	1	0
1	1	1	1
0	1	1	0
0	1	1	1
0	1	1	1
0	1	1	0
1	1	0	1
0	0	0	1
0	0	0	0
0	0	0	0
0	1	0	0
1	1	1	1
0	1	0	1
1	1	0	1
0	0	1	1

0	0	0	1
0	1	1	1
0	0	0	0
1	1	0	0
1	0	0	0
1	1	0	0
1	1	0	0
1	1	1	0
1	1	0	0
1	1	1	1
1	1	0	1
1	1	1	1
1	1	1	0
1	1	0	1
1	1	1	1
1	0	1	1
1	1	1	1
1	1	1	1
1	1	1	1
1	1	1	1
1	1	1	1
1	1	1	0
1	1	0	0
1	1	1	0
1	1	0	0
0	0	0	0
1	1	0	0
0	0	0	0
1	1	1	1
0	0	0	0
0	0	1	1
0	0	1	1
0	0	1	1
1	1	1	1
1	1	1	1
1	1	1	1
1	1	1	1
1	1	1	1
1	1	1	1
1	0	1	0
1	0	1	0
1	0	1	0
1	1	0	1
1	1	1	0
0	1	1	0
1	0	1	0
1	1	1	1

1	0	1	1
0	0	1	1
1	0	1	1
0	0	1	1
0	1	1	0
1	0	1	1
0	1	1	1
1	0	1	0
1	1	1	1
1	1	1	0
0	0	1	0
1	0	1	0
0	0	1	1
0	0	1	0
0	0	0	1
0	0	1	0
1	1	0	1
1	1	1	1
1	1	1	0
1	1	1	1
1	1	1	0
1	1	1	0
1	1	1	0
0	0	1	0
0	0	0	0
0	1	1	0
0	0	0	0
0	0	0	0
1	1	0	0
1	1	1	1
0	1	1	1
1	1	1	1
1	1	1	0
1	1	1	1
0	1	1	1
0	0	1	1
0	0	1	1
0	1	1	1
0	0	1	1
0	0	1	1
1	1	1	1
1	1	1	1
0	0	0	0
1	1	1	0
0	0	1	1
0	0	1	0
0	0	1	1

0	0	1	1
0	0	1	1
0	0	1	1
0	0	0	1
0	0	1	1
1	1	1	1
1	1	0	1
1	1	1	1
1	1	1	1
1	1	1	1
1	1	1	1

PNAS

BIOSTAT 824 Student Consortium Authors

The following authors were part of the BIOSTAT 824 Student Consortium:

Tara Al-Hashimy, Austin Allen, Nan Cen, Orlando Chen, Yongyin Chen, Yutian Chen, Tong Cheng, Yueqi Gu, Beijie Ji, Xiaohui Jiang, Fengnan Li, Peiyu Li, Yueshan Liang, Bena Liu, Coco Liu, Elisa Ma, Zhicheng Ma, Vicky Shao, Mengyao Shi, Jiang Shu, Leyi Sun, Rushi Tang, Hanyu Wang, Vivian Wang, Yuxin Wang, Krissie Wilson, Ruobing Xue, Tianyi Yang, Alison Yu, Allison Yuan, Haiqi Zhang, Vera Zhang, Yinuo Zhang

All authors in the consortium were affiliated with Duke University, NC, USA.

All authors in the consortium participated in the classroom experiment.
